# Mechanism(s) of Toxic Action of Zn^2+^ and Selenite: A Study on AS-30D Hepatoma Cells and Isolated Mitochondria

**DOI:** 10.1155/2011/387297

**Published:** 2011-07-14

**Authors:** Elena A. Belyaeva, Nils-Erik L. Saris

**Affiliations:** ^1^Laboratory of Comparative Biochemistry of Inorganic Ions, Sechenov Institute of Evolutionary Physiology and Biochemistry of Russian Academy of Sciences, Thorez Avenue 44 St. Petersburg 194223, Russia; ^2^Department of Food and Environmental Sciences, University of Helsinki, P.O. Box 56 Viikki Biocenter 1, 00014 Helsinki, Finland

## Abstract

Mitochondria of AS-30D rat ascites hepatoma cells are found to be the main target for Zn^2+^ and sodium selenite (Na_2_SeO_3_). High [mu]M concentrations of Zn^2+^ or selenite were strongly cytotoxic, killing the AS-30D cells by both apoptotic and necrotic ways. Both Zn^2+^ and selenite produced strong changes in intracellular generation of reactive oxygen species (ROS) and the mitochondrial dysfunction via the mitochondrial electron transport chain (mtETC) disturbance, the membrane potential dissipation, and the mitochondrial permeability transition pore opening. The significant distinctions in toxic action of Zn^2+^ and selenite on AS-30D cells were found. Selenite induced a much higher intracellular ROS level (the early event) compared to Zn^2+^ but a lower membrane potential loss and a lower decrease of the uncoupled respiration rate of the cells, whereas the mtETC disturbance was the early and critical event in the mechanism of Zn^2+^ cytotoxicity. Sequences of events manifested in the mitochondrial dysfunction produced by the metal/metalloid under test are compared with those obtained earlier for Cd^2+^, Hg^2+^, and Cu^2+^ on the same model system.

## 1. Introduction

Zinc (Zn) and selenium (Se) are essential microelements with several important biological functions; among them are the maintenance of tissue integrity and function, proliferation, regulation of cellular thiol redox state, stabilization of intracellular environment, and protection against various stressors [1–4]. Despite Zn and Se are dietary nutrients for all mammalian species, an excess of these trace elements is harmful that produces the strong toxicity both to animals and humans and to the cultured cells. As well known now, the biological effects of Zn and Se are strictly dose dependent, with antioxidant properties at low concentrations and potent prooxidant effects at moderate to high concentrations. In particular, both Zn^2+^ and different Se compounds, especially sodium selenite (Na_2_SeO_3_), exhibit strong prooxidant effects on cells of different types that underlie, as generally accepted, for their anticancer activity [5–8]. However, the exact molecular mechanisms of toxic action of Zn^2+^ and selenite, namely, role of mitochondria and reactive oxygen species (ROS), the involvement of mitochondrial electron transport chain (mtETC), as well as sequences of events manifested in the mitochondrial dysfunction and cytotoxicity are not well understood up to date. So, using AS-30D rat ascites hepatoma cells cultivated in vitro, that is, the same model system as we applied in our previous works under studying the role of mitochondrial dysfunction in heavy-metal induced cytotoxicity [9, 10], we aimed to elucidate molecular mechanism(s) of toxicity of Zn^2+^ and selenite and compared them with those obtained earlier for Cd^2+^, Hg^2+^, and Cu^2+^. The findings shown on the intact cells correlate well with our data found on isolated rat liver mitochondria in the present work and before [11]. Portions of this work were presented previously in an abstract form and as a part of a published lecture [12, 13].

## 2. Materials and Methods

### 2.1. Chemicals

Carbonyl cyanide 3-chlorophenylhydrazone (CCCP), oligomycin, valinomycin, bongkrekic acid, ZnCl_2_, and Na_2_SeO_3_ were purchased from Sigma (St. Louis, MO). Propidium iodide, 5,5′,6,6′-tetrachloro-1,1′,3,3′-tetraethylbenzimidazolcarbocyanine iodide (JC-1), and 2′,7′-dichlorodihydrofluorescein diacetate (DCFH_2_-DA) were from Molecular Probes (Eugene, OR). Cyclosporine A (CsA) was from Novartis (Basel, Switzerland). RPMI-1640 medium and all cell culture supplies were products of GIBCO BRL (Grand Island, NY). Other chemicals used were of analytical reagent grade.

### 2.2. Cell Culture

Rat ascites hepatoma cells (AS-30D), kindly provided by Dr. Antonio Villalobo (Institute for Biomedical Research, National Research Council and Autonomous University of Madrid, Spain), were maintained in RPMI-1640 medium containing 20 mM Hepes-NaOH (pH 7.4) and supplemented with 2 mM L-glutamine, 10% fetal calf serum, and 40 *μ*g/mL gentamycin at 37^ °^C in a humidified atmosphere of 5% CO_2_ in air, essentially as described previously [9, 14]. The cells were seeded at a density of 0.5 × 10^6^ cells/mL and used after being cultured overnight. ZnCl_2_ and Na_2_SeO_3_ were dissolved in distilled water to a 10 mM stock solution, and this was further diluted with the medium or phosphate buffered saline (PBS) to the desired concentrations. The following concentrations of the metal/metalloid were used: 10, 50, 100, 250, and 500 *μ*M—for Zn^2+^ and 0.1, 0.5, 1, 5, 10, and 50 *μ*M—for selenite.

### 2.3. Preparation of Rat Liver Mitochondria

Rat liver mitochondria were prepared by differential centrifugation after homogenization in a mannitol-sucrose medium containing 1 mM EGTA ([ethylenebis(oxyethylene-nitrilo)]tetraacetic acid) and 0.5% BSA (bovine serum albumin) according to [15]. Mitochondria were washed two times using a medium without EGTA and BSA. Finally they were suspended in a medium containing 220 mM mannitol, 70 mM sucrose, 10 mM Hepes/Tris, and pH 7.4. The most of experiments was repeated also on the mitochondria, which were isolated using a homogenization medium containing 250 mM sucrose, 10 mM Tris-HCl, pH 7.4, and 0.5 mM EGTA-Tris, while the EGTA was omitted in the washing medium [16]. The results obtained in both cases are the same. Protein content was measured by the Biuret method with BSA as standard.

### 2.4. Cytotoxicity Assays

Cell viability was assayed by the trypan blue exclusion test and expressed as percentage of cells that did not take up the dye. To determine the proportion of apoptosis, the cells were cultivated in RPMI 1640 medium without (control) or with given concentration of Zn^2+^ or selenite for 24 h, stained with propidium iodide and analyzed by flow cytometry as the sub-G_1_ fraction [17]. About 10^4^ cells were used for each run; for more details see also [9, 14]. Flow cytometry was performed using FACS Calibur instrument (FL-2 channel) with Cell-Quest software (Becton Dickinson, San Jose, CA). It seems important to add that although discrimination between cells undergoing necrosis and apoptosis is not always straightforward, we have assumed in this study, in accordance with similar investigations by other authors (see, e.g., [18]), that the trypan blue-exclusion assay, depicting the loss of plasma membrane integrity, corresponds with a certain approximation to necrosis, whereas DNA fragmentation assayed by propidium iodide staining and quantification of the resulting sub-G_1_ fraction were used as a commonly accepted test for apoptosis [17].

### 2.5. Mitochondrial Function Assays

#### 2.5.1. AS-30D Rat Ascites Hepatoma Cells

ROS production was measured with the oxidation-sensitive fluorescent probe DCFH_2_-DA by flow cytometry, using FL-1 channel and 10,000 cells for each run, as described previously [9, 14]. ROS generation was calculated as the geometrical mean of the total green fluorescence of the oxidation product, 2′,7′-dichlorofluorescein (DCF). Changes in mitochondrial transmembrane potential (ΔΨ_mito_) were monitored after staining with the lipophilic cationic probe JC-1, using channels FL-1 and FL-2 of the flow cytometer [19]. Respiration of the cells was measured polarographically using Clark-type oxygen electrode (Yellow Springs Instruments, Yellow Springs, OH) in a thermostatic water-jacketed vessel at 37°C. Total volume was 1.0 mL and the amount of cells was 10^7^. The respiratory buffer contained PBS supplemented with 5 mM glutamine and 5 mM pyruvate. Under these conditions, oxygen uptake by the cells was used to sustain a steady-state level of ΔΨ_mito_ by compensating for the proton leak and ATP synthesis by mitochondrial ATP synthase (F_1_F_o_-ATPase) that designated as the “steady-state” respiration. Addition of the inhibitor of F_1_F_o_-ATPase, oligomycin, decreased cell respiration, leaving only that portion of oxygen uptake, which compensated for the proton leak (“resting respiration”). The chemical protonophore CCCP was added to induce maximal rate of respiration (“uncoupled respiration”, limited only by efficiency of the respiratory chain [20]) and to test the participation of the mtETC in the Zn^2+^- or selenite-induced cell damage. The concentration of oligomycin and CCCP, when added, was 1 *μ*g/mL and 1 *μ*M, respectively.

#### 2.5.2. Rat Liver Mitochondria

Measurements of oxygen consumption by isolated mitochondria were conducted polarographically using a Clark-type electrode as before [16]. The ΔΨ_mito_ was estimated using a tetraphenylphosphonium (TPP^+^) selective electrode, the distribution of this probe depending on the membrane potential. Besides, selective electrodes were used to record changes in [K^+^] and [Ca^2+^]. For composition of media and experimental details, see figure legends.

### 2.6. Statistical Analysis

The results shown are representative or average from a minimum of three independent experiments. Data are expressed as mean values ±SE; statistical significance was analyzed, using Student's *t*-test with *P* < 0.05 assumed as the significance threshold.

## 3. Results and Discussion

### 3.1. AS-30D Rat Ascites Hepatoma Cells

#### 3.1.1. Zn^2+^ and Selenite Action on Cell Viability

**Figure 1:**
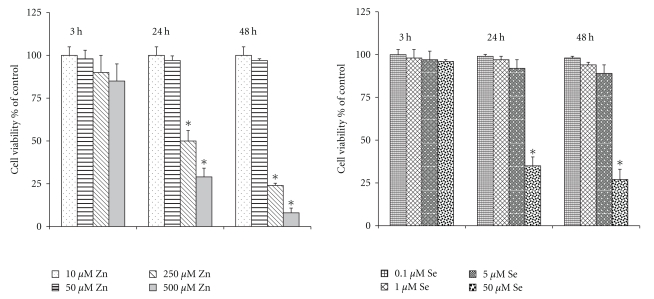
Time- and dose-dependent action of Zn^2+^ and selenite on AS-30D cell viability assayed by the trypan blue exclusion test. Time of incubation used was 3, 24, and 48 h. The results are expressed in % to corresponding control and presented as mean values of four independent experiments ±SE. *P < 0.05.

**Figure 2:**
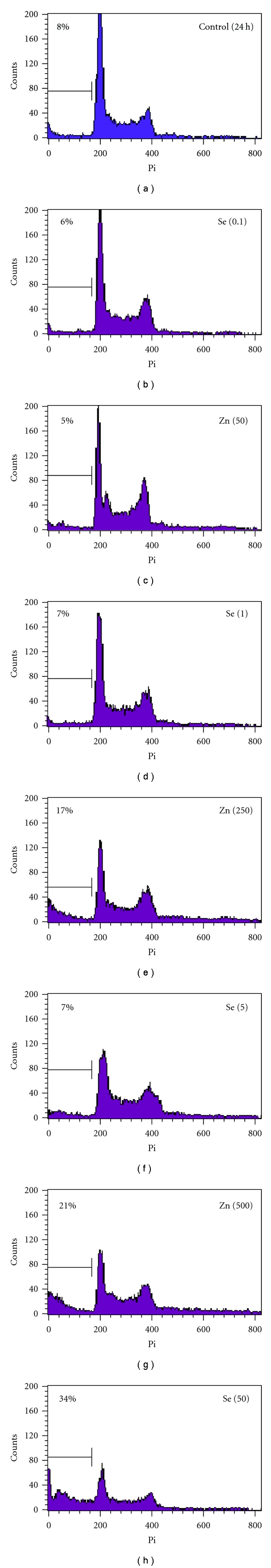
Induction of apoptosis in AS-30D cells by different concentrations of Zn^2+^ and selenite. The cells were cultivated in RPMI 1640 medium without (control) or with the indicated concentration (in *μ*M) of Zn^2+^ and selenite for 24 h, stained with propidium iodide, and analysed by flow cytometry. Percentage of the sub-G_1_ fraction, characteristic for apoptotic cells, is indicated in the upper left corner of each panel. A typical experiment out of at least three independent ones for each compound is shown.

At first, we studied dose and time dependence of Zn^2+^ and selenite action on AS-30D cell viability. We found that high concentrations of selenite (50 *μ*M) and Zn^2+^ (250 and 500 *μ*M) killed AS-30D cells time-dependently by both necrotic and apoptotic ways (see Figures 1 and 2, correspondingly). At the same time, 10–50 *μ*M of Zn^2+^ and 0.1–5 *μ*M of selenite evoked neither necrotic (Figure 1) nor apoptotic (Figure 2) death of AS-30D cells at used durations of incubation. So, the main objective of the next experiments was to underscore molecular mechanisms of cytotoxicity of high *μ*M concentrations of Zn^2+^ and selenite. 

In this context, we investigated the action of several modulators of mitochondrial permeability transition (MPT) pore (i.e., a nonselective high-conductance inner mitochondrial membrane channel of unknown structure, the opening of which enables free passage into the mitochondria of molecules of <1.5 kDa including protons that are found to be involved in many pathological conditions and cell death of different types [21–24]) against Zn^2+^- and selenite-induced injury of AS-30D cells. We found that CsA, a potent pharmacological MPT pore inhibitor taken in concentration of 1 *μ*M that did not produce any significant effect on AS-30D cells per se, partly prevented the cell death measured after 24 h exposure of the cells to high selenite or Zn^2+^(Table 1). 

It seems important to say that previously we compared toxic action of *μ*M concentrations of Cd^2+^, Hg^2+^, and Cu^2+^ (i.e., 10, 50, 100, and 500 *μ*M) on rat ascites hepatoma AS-30D cells and found that the toxicity of these three metal ions decreased from Hg^2+^ (most toxic) to Cu^2+^ (least toxic) [10]. Hg^2+^ and Cd^2+^ produced a high percentage of cell death by both necrosis and apoptosis, whereas Cu^2+^ at concentrations up to 500 *μ*M was weakly effective. All the metals produced significant changes in the mitochondrial function and in intracellular ROS generation. Moreover, our data showed that increased ROS level alone was not sufficient to induce apoptotic and/or necrotic decay of AS-30D cells. In the case of Cd^2+^ and Hg^2+^, additional factor(s) must have been present that was/were responsible for their cytotoxic action; most likely, it was the blockage of the mitochondrial respiratory chain [10]. We revealed also that the Cd^2+^-produced cytotoxicity was accompanied by increased ROS formation at the mitochondrial respiratory chain complex III level and the MPT pore induction [9].

#### 3.1.2. Zn^2+^and Selenite Action on ΔΨ_mito_

**Figure 3:**
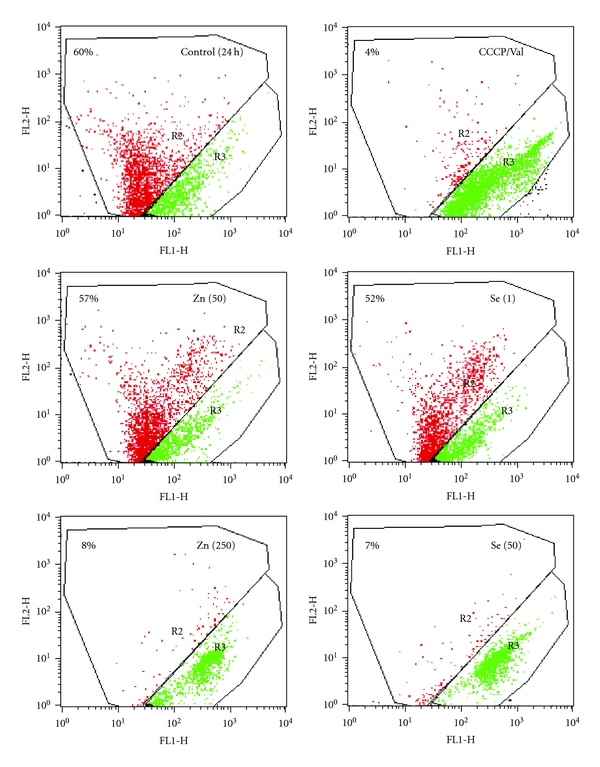
Action of different concentrations of Zn^2+^ and selenite on the mitochondrial transmembrane potential after 24 h incubation with AS-30D cells. JC-1 is a cell-penetrating dye that accumulates within mitochondria maintaining high ΔΨ_mito_ and changes its emission fluorescence from green to red that can be followed by flow cytometry. The percentage of cells with red (R2) and green (R3) JC-1 fluorescence, reflecting high and low ΔΨ_mito_, respectively, was estimated; R2 is indicated in the upper left corner of each panel. The protonophoric uncoupler CCCP plus the potassium ionophore valinomycin, acting together to completely collapse ΔΨ_mito_, have always been used as the “positive” control. A typical experiment out of at least three independent ones is shown.

To further elucidate the mechanisms underlying the cytotoxicity of high concentrations of Zn^2+^ and selenite, we studied the influence of this metal/metalloid on ΔΨ_mito_. As seen from Table 2, after 3 h incubation of AS-30D cells with 50 *μ*M of selenite, the significant decline in ΔΨ_mito_ (approximately on 30% compared to control) was observed. During the same time, 500 *μ*M of Zn^2+^ produced a more than twofold decrease in ΔΨ_mito_ compared to the control. After 24 h incubation, both high Zn^2+^ and selenite caused the severe ΔΨ_mito_ loss of AS-30D cells (Table 2 and Figure 3). It is worthy of note that low Zn^2+^ (50 *μ*M) and selenite (1 *μ*M) did not produce any significant changes in ΔΨ_mito_ of AS-30D cells even after 24 h incubation with the cells (Figure 3). 

As to Cd^2+^, Hg^2+^, and Cu^2+^ [10], when measuring ΔΨ_mito_ in the intact AS-30D cells, we found that 50 *μ*M Hg^2+^ produced a complete collapse of ΔΨ_mito_ already after 30 min and 10 *μ*M Hg^2+^produced the strong ΔΨ_mito_ decrease (about 80% compared to control) already after 3 h exposure with the cells. During that short incubation time, Cd^2+^ exerted a dose-dependent effect that became statistically significant only at concentrations of 100 *μ*M and 500 *μ*M. Cu^2+^ had a smaller inhibitory effect on ΔΨ_mito_ after 3 h, which was still statistically insignificant for 100 *μ*M Cu^2+^ after 24–48 h, while 500 *μ*M Cu^2+^ strongly depressed ΔΨ_mito_ after 48 h. 

#### 3.1.3. Zn^2+^ and Selenite Action on Cell Respiration

To next clarify the mechanism(s) of mitochondrial dysfunction involved in the harmful effects of Zn^2+^ and selenite, we examined their action on the cell respiration. As shown in Table 3, the uncoupled respiration rate of AS-30D cells, that is in the presence of CCCP (see Section 2), began to decline after 3 h incubation with 50 *μ*M of selenite, and after 48 h its magnitude was 20% of the control. In the case of high Zn^2+^, the uncoupled respiration rate of the cells decreased more than twofold already after 3 h, and was completely inhibited after 48 h incubation with 500 *μ*M of Zn^2+^ (Table 3). At the same time, low concentrations of Zn^2+^ (10, 50, and even 100 *μ*M) and selenite (up to 5 *μ*M) did not affect the uncoupled respiration rate of AS-30D cells at all incubation times under study, while 10 *μ*M of selenite after 48 h exposure with the cells decreased significantly the uncoupled respiration rate which was 80% of the control (data not shown). It is important to say also that 50 *μ*M of selenite evoked a significant (30%) stimulation of the resting respiration rate (i.e., in the presence of oligomycin, see Section 2) of AS-30D cells after 3 h incubation, whereas high Zn^2+^ (250 and 500 *μ*M) significantly inhibited the cell respiration in the resting state, starting already from 3 h treatment. All other concentrations of Zn^2+^ and selenite under test did not produce significant effects on the resting state respiration rate of AS-30D cells. It should be noted that in the case of high Zn^2+^ only the inhibitory action on the resting respiration of AS-30D cells was obtained together with the strong inhibitory effect on the uncoupled respiration rate, pointing to a blockage of the mitochondrial respiratory chain.

In turn, before [10], we found that after 3 h incubation of AS-30D cells with 10 *μ*M Hg^2+^ the resting state respiration was slightly but significantly increased, whereas the uncoupled respiration remained unaffected, pointing to a weak uncoupling effect of that low concentration of the metal. In contrast, at 50 *μ*M Hg^2+^, all three values (i.e., the steady-state respiration, the resting respiration, and the uncoupled respiration) were strongly depressed, indicating a potent inhibitory effect on the respiratory chain. With Cd^2+^, a decrease by about 30% of the uncoupled respiration was observed at 100 *μ*M concentration and some inhibitory effect could be seen already at 50 *μ*M concentration. Further increasing Cd^2+^ concentration to 500 *μ*M increased the inhibitory effect. In contrast, Cu^2+^ had no inhibitory effect even at 500 *μ*M concentration after 3 h, but exerted a weak uncoupling effect, as manifested by an increase of both steady-state and resting respiration rates. After 24 h incubation of the cells with the corresponding heavy metal, 50 *μ*M Hg^2+^ and 50 *μ*M Cd^2+^ produced a practically complete depression of cellular respiration, while 50 *μ*M Cu^2+^ exerted a pronounced uncoupling effect; moreover, even after 48 h 50–100 *μ*M Cu^2+^ did not inhibit the cell respiration, whereas 500 *μ*M Cu^2+^ decreased it to 70% or below.

#### 3.1.4. Zn^2+^ and Selenite Action on Intracellular ROS Production

**Figure 4:**
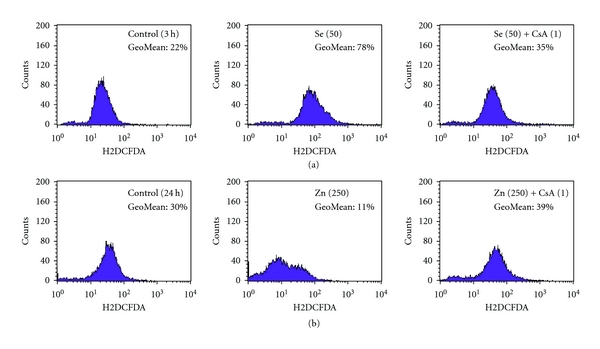
Action of cyclosporine A on changes in intracellular ROS formation produced by high Zn^2+^ and selenite in AS-30D cells. (a) Se: 50 *μ*M; 3 h; (b) Zn: 250 *μ*M; 24 h. The cells were cultivated in RPMI 1640 medium without (control) or with the indicated concentration (in *μ*M) of selenite and Zn^2+^ for 3 h and 24 h, respectively, in the absence or presence of 1 *μ*M CsA. The overall ROS production was calculated as the geometric mean of total green fluorescence of the oxidation product of DCFH_2_ and is indicated in the upper right corner of each panel; for other details, see Section 2. A typical experiment out of at least three independent ones for each compound is shown.

As known, intracellular ROS production can be an important indicator of cytotoxicity of compounds under study. As shown in Table 4, 50 *μ*M of selenite enhanced in three times the intracellular ROS generation already after short incubation times (50 min and 3 h), while after 24–48 h they evoked the sharp reduction of the ROS production compared to control. In contrast, high concentrations of Zn^2+^ under test, namely, 250 and 500 *μ*M, did not produce any significant changes in the ROS generation after 50 min of incubation with the AS-30D cells. Nevertheless, high Zn^2+^ increased moderately the intracellular ROS formation after 3 h and decreased their production in a half (compared to control) after 24–48 h of incubation with the cells (Table 4). Low Zn^2+^ (50 *μ*M) and selenite (up to 5 *μ*M) did not change the ROS generation of AS-30D cells after all durations of incubation used. We found also that the intracellular ROS generation changes, observed after incubation of AS-30D cells with high Zn^2+^ or selenite, were attenuated by CsA (Figure 4). 

It should be mentioned that in our previous work on AS-30D cells [10] we showed that Cu^2+^ induced an early and sharp increase of the intracellular ROS generation. In particular, Cu^2+^ at the range of 100–500 *μ*M induced only stimulation of the ROS formation that started as early as after 30 min of incubation with the cells; however, the ROS production decreased to the level of the control after 48 h incubation of the cells with 500 *μ*M of Cu^2+^. The action of Hg^2+^ and Cd^2+^ on the ROS formation was biphasic. They stimulated ROS generation within the cells at low concentrations and at short incubation times but decreased the ROS generation at higher concentrations and at longer incubation.

### 3.2. Rat Liver Mitochondria

**Figure 5:**
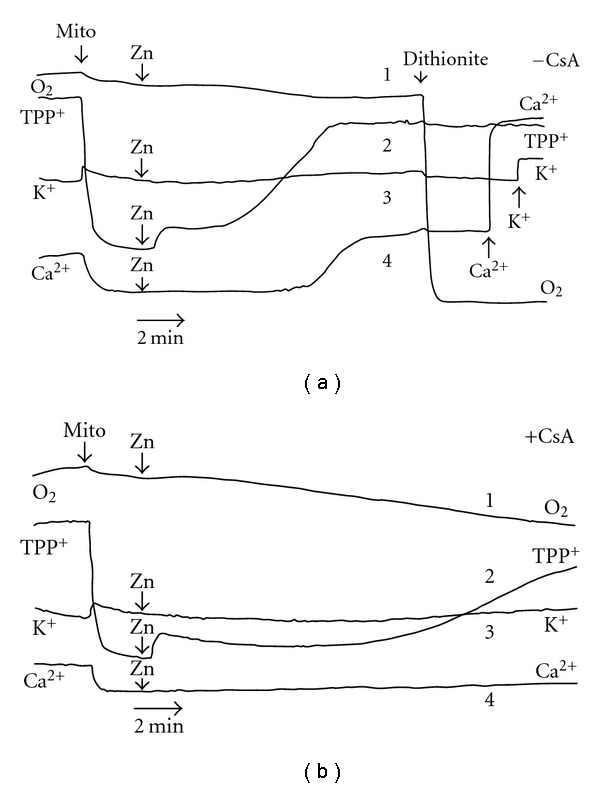
Simultaneous recordings of four mitochondrial parameters (respiration, ΔΨ_mito,_ K^+^, and Ca^2+^ fluxes) with the help of O_2_, TPP^+^, K^+^, and Ca^2+^-selective electrodes after treatment of isolated RLM with Zn^2+^ in the absence (a) or presence of CsA (b). Mitochondria (1 mg protein/mL) were incubated at room temperature in a medium containing 120 mM NaCl, 2 mM NaH_2_PO_4_, 10 mM HEPES (pH 7.4), 5 mM Glu, and 5 mM Mal. The additions of Zn^2+^ (5 *μ*M), K^+^(100 *μ*M), Ca^2+^(200 *μ*M), and dithionite are indicated by arrows. [CsA] was 1 *μ*M. 20 *μ*M of Ca^2+^were present in the assay medium from the beginning of experiment. Downward deflection indicates decrease in [O_2_], [TPP^+^], [K^+^_free_], and [Ca^2+^_free_] in the medium. The results are representative for a series of two independent experiments.

**Figure 6:**
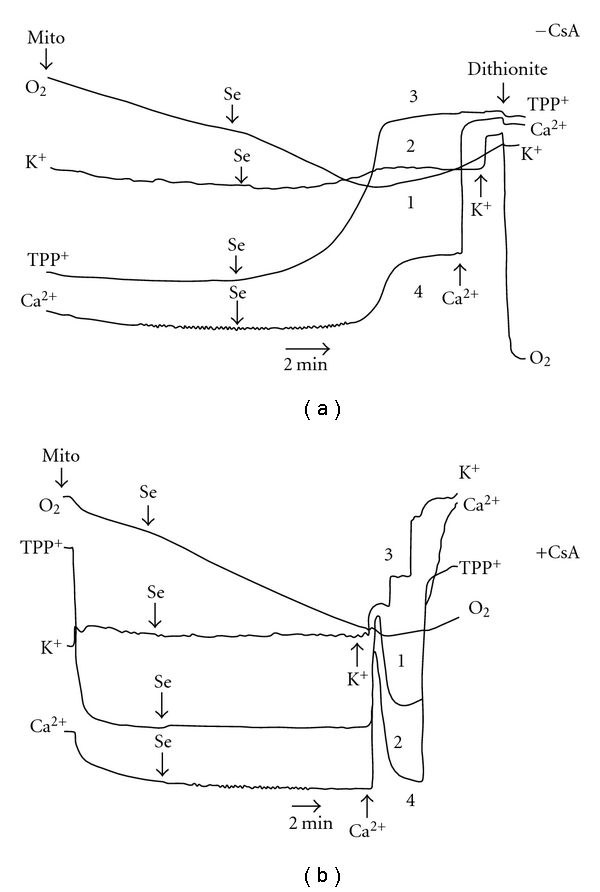
Simultaneous recordings of four mitochondrial parameters (respiration, ΔΨ_mito_, K^+^, and Ca^2+^ fluxes) with the help of O_2_, TPP^+^, K^+^, and Ca^2+^-selective electrodes after treatment of isolated RLM with sodium selenite in the absence (a) or presence of CsA (b). The additions of Na_2_SeO_3_ (5 *μ*M) are indicated by arrows. The remainder is as in Figure 5.

To further understand the molecular mechanism(s) of mitochondrial dysfunction produced by the metal/metalloid under test, we compared the action of Zn^2+^ with effects of selenite on isolated rat liver mitochondria used as a model system. With the help of O_2_, TPP^+^, K^+^, and Ca^2+^-selective electrodes, the simultaneous monitoring of four bioenergetics parameters—respiration, ΔΨ_mito,_ K^+^, and Ca^2+^ fluxes—was conducted in the presence of the metal/metalloid under study and different mitochondrial effectors to underscore the cause/consequence relationships underlying the mitochondrial dysfunction (Figures 5 and 6) 

We found that in NaCl respiratory assay medium where K^+^ was replaced by Na^+^ in order to monitor K^+^ fluxes in and out of mitochondria (for the exact medium content, see legend to Figure 5), the ΔΨ_mito_ decrease, K^+^ and Ca^2+^ release, and the respiratory disturbance produced by low [Zn^2+^] were strongly depressed by CsA, a potent inhibitor of the MPT pore (Figure 5). However, even in the presence of CsA in the assay medium, there was still a slow dissipation of Ψ_mito_ after Zn^2+^ addition (Figure 5(b), trace 2). As evident also, Ca^2+^release from the mitochondria was the last event among observed in the presence of Zn^2+^ (Figure 5(a), trace 4). Previously in KCl respiratory assay medium, we observed the similar changes in ΔΨ_mito_ evoked by Zn^2+^ (measured with the help of Rh123), which again were only partially sensitive to CsA [11]. Before we found also that in the KCl medium Zn^2+^ induced a sharp oxidation of pyridine nucleotides (PN) that was inhibited by CsA, the protective action of which, however, was overridden by increase of Zn^2+^ load. In addition, the mitochondrial swelling produced by Zn^2+^ in the KCl and sucrose media was retarded by CsA and other MPT inhibitors [11].

The action of Zn^2+^ on the mitochondrial function resembles very much the Cd^2+^ effects studied by us before ([11, 13, 16, 24, 25], see also Figure 8 here), namely, the respiratory dysfunction, the ΔΨ_mito_ loss, K^+^ and Ca^2+^ release, changes in the mitochondrial PN redox state, and matrix swelling produced by Cd^2+^ were strongly inhibited by CsA. Nevertheless, even in the presence of CsA in the assay medium, a slow dissipation of the ΔΨ_mito_ by Cd^2+^ took place (Figure 8(b), trace 2). Moreover, again as in the case of Zn^2+^, the Ca^2+^ release was the last event among observed after Cd^2+^ addition; besides, the defense exhibited by CsA against the harmful effects of Cd^2+^ was eliminated by increase of the heavy metal load. 

**Figure 7:**
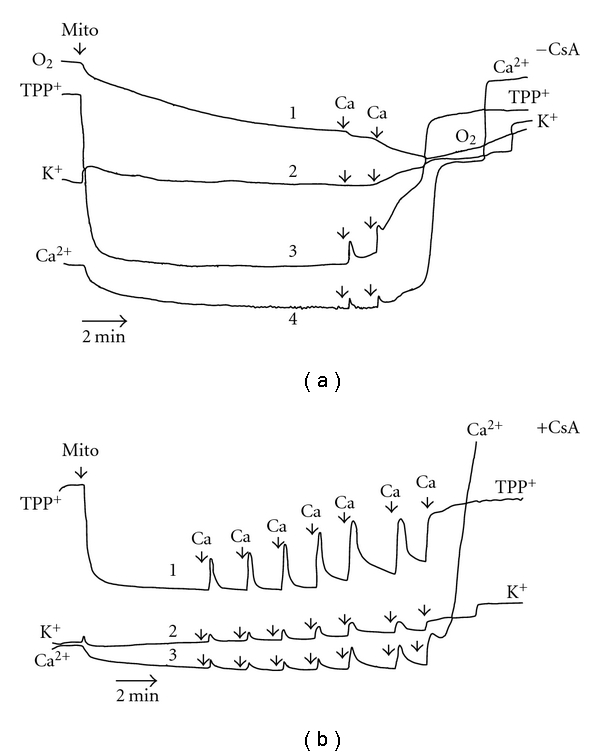
Simultaneous recordings of four mitochondrial parameters (respiration, ΔΨ_mito,_ K^+^, and Ca^2+^ fluxes) with the help of O_2_, TPP^+^, K^+^, and Ca^2+^-selective electrodes after treatment of isolated RLM with Ca^2+^ in the absence (a) or presence of CsA (b). The additions of Ca^2+^ (50 *μ*M) are indicated by arrows. The remainder is as in Figure 5.

**Figure 8:**
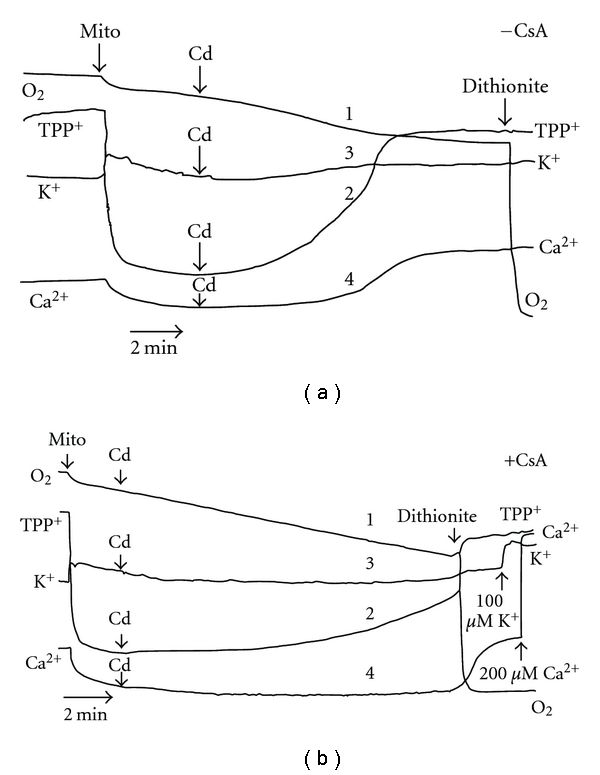
Simultaneous recordings of four mitochondrial parameters (respiration, ΔΨ_mito,_ K^+^, and Ca^2+^ fluxes) with the help of O_2_, TPP^+^, K^+^, and Ca^2+^-selective electrodes after treatment of isolated RLM with Cd^2+^ in the absence (a) or presence of CsA (b). The additions of Cd^2+^ (5 *μ*M) are indicated by arrows. The remainder is as in Figure 5.

In the case of selenite, we found that not only K^+^ or Ca^2+^ release (Figure 6) but also the ΔΨ_mito_ loss induced by selenite pulses both in the NaCl (Figure 6) and in the KCl [11] assay media was completely inhibited by CsA. The same was true for the Ca^2+^-induced ΔΨ_mito_ dissipation (Figure 7). However, a strong sustained activation of the basal mitochondrial respiration rate found in the presence of selenite was only partially sensitive to CsA (Figure 6(a) and 6(b), traces 1). As seen also from Figure 6, CsA restored the Ca^2+^ uptake capacity disturbed by the selenite treatment (Figure 6(b), trace 4). It should be noted that the significant sustained stimulation of the resting respiration of the mitochondria energized by Glu *plus *Mal (i.e., mtETC complex I substrates) in the presence of selenite was observed in the KCl assay medium as well; in addition, we found before that in this medium selenite decreased both the respiration in St 3 and the DNP-stimulated respiration [11]. As we have shown also, the mitochondrial swelling induced by selenite in the KCl medium in the presence of Glu *plus *Mal was only partially susceptible to CsA in opposite to the Ca^2+^-induced one which was completely depressed by CsA. It is worthy to note that on this type of respiratory substrates, Ca^2+^ produced only a transient stimulation of the resting respiration followed by the strong inhibition of the mitochondrial respiration both in this medium (see, for example, [26] and references therein) and in the NaCl assay buffer (Figure 7(a), trace 1). Besides, as we found previously [11], the influence of selenite on the PN redox status differed strongly not only from those of Zn^2+^ or Cd^2+^ but from that of Ca^2+^ as well. In particular, after serial addition of 5 *μ*M pulses of selenite in the KCl assay medium, there was no strong and rapid decrease in the PN autofluorescence (eliminated by CsA supplement into the medium) as it was observed after addition of the mentioned above metal pulses (5 *μ*M—for Zn^2+^or Cd^2+^ and 50 *μ*M—for Ca^2+^) but there was the moderate and continuous PN oxidation which was only slow down by CsA under used experimental conditions. 

As to about action of Hg^2+^ or Cu^2+^ on isolated rat liver mitochondrial function, we found in our previous works [27, 28] that under the same conditions PN oxidation, respiratory dysfunction, and ΔΨ_mito_ decrease produced by these heavy metals were insensitive or weakly sensitive to CsA. Besides, CsA and other MPT pore effectors, including several mtETC inhibitors affected differently the mitochondrial swelling induced by the metals/metalloid under test [11].

### 3.3. Concluding Remarks

In the present study, we have found that mitochondria of AS-30D rat ascites hepatoma cells are the main target not only for Cd^2+^, Hg^2+^, and Cu^2+^ as we obtained before [9, 10] but also for Zn^2+^ and sodium selenite. High *μ*M concentrations of Zn^2+^ or selenite were strongly cytotoxic, killing the AS-30D cells by both apoptotic and necrotic ways (Figures 1 and 2). Both Zn^2+^ and selenite produced strong changes in the intracellular ROS generation (Table 4 and Figure 4) and the mitochondrial dysfunction via the mtETC disturbance (Table 3), the membrane potential dissipation (Table 2 and Figure 3), and the MPT pore opening (Table 1 and Figure 4). The significant distinctions in toxic action of Zn^2+^ and selenite on AS-30D cells were revealed as well. In particular, selenite induced a much higher intracellular ROS level (the early event) compared to Zn^2+^ but a lower membrane potential loss and a lower decrease of the uncoupled respiration rate of the cells, whereas the mtETC disturbance was the early and critical event in the mechanism of Zn^2+^ cytotoxicity. 

Importantly, the findings obtained on the intact cells correlate well with our data shown on isolated mitochondria. In particular, we found that the stimulation of the basal respiration of the isolated rat liver mitochondria produced by selenite was only partially depressed by CsA (Figure 6). In accordance with these results our data revealed on AS-30D cells, namely, there were the stimulation of the resting state respiration rate and the significant decrease of the ΔΨ_mito_ found already after 3 h incubation of the cells with high selenite that indicates the uncoupling effect of the metalloid at early times of incubation. Besides, the rapid and potent burst of the intracellular ROS formation found in the presence of high selenite after 3 h incubation with AS-30D cells was partially depressed by CsA (Figure 4(a)). 

It should be mentioned that up to date the role of Zn and Se compounds in prevention and possible treatment of cancer still remains obscure due to their complex interactions with cells and tissues. Nevertheless, a lot of evidence indicates direct toxicity and proapoptotic activity towards malignant cells of externally supplemented Zn^2+^ [6, 29–31] or selenite [32–36]. The involvement of mitochondria and oxidative stress in different types of cell death produced by Zn^2+^ and selenite was found before [37–45]; however, several authors still argue against the direct participation of ROS in cytotoxicity mechanisms of Zn^2+^ and selenite [8, 30, 35, 46, 47]. It is worthy to say that in previous publications the participation of the “classical” MPT pores (i.e., Ca^2+^-dependent and CsA-sensitive [21–23]) in mechanism(s) of toxic action of both selenite [11, 34, 36, 40, 47–50] and Zn^2+^ [6, 11, 51–56] was suggested; however, at the moment it is under debate in some aspects [56–59]. In particular, there is recent evidence indicating a possible involvement of unregulated MPT pores (i.e., Ca^2+^-independent and CsA-insensitive, [22, 60]) in mechanisms of toxicity of Se compounds [59]. Despite the existence of data about preventive action of the MPT pore inhibitors, CsA and bongkrekic acid, on ΔΨ_mito_ dissipation and cytochrome c or AIF release produced by selenite in different types of cells, in the literature in the field we could not find evidence on action of CsA on intracellular ROS generation changes in the presence of this metalloid. Importantly, in the present work we conducted, for the first time, a thorough comparative study of Zn^2+^ and selenite effects on cellular respiration. Thus, our findings are not only in a good accordance with data found by other workers on different cell lines during the years, but give new important information about the molecular mechanism(s) underlying Zn^2+^- and selenite-induced mitochondrial dysfunction and cytotoxicity, in support to a recent series of publications on the issue [61–68]. It should be stressed also that a comprehensive comparison of toxic effects of the metals/metalloid on isolated mitochondria and on the same cell line under the same conditions conducted herein revealed the significant similarities and distinctions in the mechanisms of their action and gave a key to better understanding of the role of the mitochondrial dysfunction in cell death, pointing to a possible combined use of these compounds in anticancer therapy. This issue is now under study in our group.

## Figures and Tables

**Table 1:** Action of cyclosporine A on AS-30D cell death produced by high concentrations of Zn^2+^ and selenite.

Treatment (24 h)	50 *μ*M Se	250 *μ*M Zn	500 *μ*M Zn
none	27 ± 3	31 ± 5	17 ± 6
+ CsA (1 *μ*M)	41 ± 1*	55 ± 5**	30 ± 6**

The cell viability assayed by the trypan blue exclusion test is expressed in % to untreated control. Mean ±SE; *n* = 4 independent experiments; *P < 0.05 compared to selenite-treated control; **P < 0.05 compared to Zn(II)-treated control.

**Table 2:** Effects of high concentrations of Zn^2+^ and selenite on mitochondrial transmembrane potential of AS-30D cells monitored by flow cytometry after staining the cells with the lipophilic cationic probe JC-1.

Time	50 *μ*M Se	250 *μ*M Zn	500 *μ*M Zn
3 h	67 ± 11*	55 ± 9*	46 ± 2*
24 h	14 ± 3*	13 ± 1*	12 ± 4*

The percentage of cells with red JC-1 fluorescence, reflecting high ΔΨ_mito_ (expressed in % to the corresponding control), is presented; the percentage of cells with high ΔΨ_mito_ in untreated control is accepted as 100%. Mean ±*SE*; *n* = 3 independent experiments; *P < 0.05 compared to untreated control.

**Table 3:** Effects of high concentrations of Zn^2+^ and selenite on the uncoupled respiration of AS-30D cells. The rate of uncoupled respiration (in the presence of CCCP) of control cells is accepted as 100%.

Time	50 *μ*M Se	250 *μ*M Zn	500 *μ*M Zn
3 h	86.4 ± 4.6*	48.4 ± 7.2*	28.3 ± 10.6*
24 h	n.d.	31.0 ± 5.0*	10.7 ± 0.5*
48 h	20.8 ± 6.5*	11.3 ± 1.3*	1.9 ± 0.3*

Rate of oxygen uptake is presented in percentage of the maximal (fully uncoupled) respiration of the control cells. Mean ± SE; *n* = 3 independent experiments; *P < 0.05 compared to untreated control. For other details, see Section 2; n.d., not determined.

**Table 4:** Time-dependent effects of high concentrations of Zn^2+^ and selenite on ROS formation by AS-30D cells measured by flow cytometry using DCFH_2_-DA as ROS-sensitive probe.

Medium	Time	DCF fluorescence (arbitrary units)			
		Control	50 *μ*M Se	250 *μ*M Zn	500 *μ*M Zn
PBS	50 min	32.8 ± 6.3	90.9 ± 3.1*	38.5 ± 2.2	29.2 ± 9.5
RPMI	3 h	21.1 ± 1.1	75.6 ± 2.5*	31.6 ± 1.5*	32.1 ± 1.8*
	24 h	25.8 ± 4.3	4.2 ± 0.8*	12.0 ± 1.0*	n.d.
	48 h	21.7 ± 4.7	7.3 ± 0.7*	11.8 ± 0.5*	n.d.

The overall ROS production is shown as the geometric mean (±SE, *n* = 3) of total green fluorescence of the oxidation product of DCFH_2_. *P < 0.05 compared to untreated control; for other details, see Section 2; n.d., not determined.

## Abbreviations

ROS: Reactive oxygen species
mtETC: Mitochondrial electron transport chain
CCCP: Carbonyl cyanide 3-chlorophenylhydrazone
JC-1: 5,5′,6,6′-tetrachloro-1,1′,3,3′-tetraethylbenzimidazolcarbocyanine iodide
DCFH2-DA: 2′,7′-dichlorodihydrofluorescein diacetate
CsA: Cyclosporine A
PBS: Phosphate Buffered Saline
EGTA: [Ethylenebis(oxyethylene-nitrilo)]tetraacetic acid
BSA: Bovine serum albumin
DCF: 2′,7′-Dichlorofluorescein
ΔΨ_mito_: Mitochondrial transmembrane potential
TPP^+^: Tetraphenylphosphonium
MPT: Mitochondrial permeability transition
PN: Pyridine nucleotides.
